# Comprehensive Bioinformatics Analysis Identifies *POLR2I* as a Key Gene in the Pathogenesis of Hypertensive Nephropathy

**DOI:** 10.3389/fgene.2021.698570

**Published:** 2021-08-05

**Authors:** Shilong You, Jiaqi Xu, Boquan Wu, Shaojun Wu, Ying Zhang, Yingxian Sun, Naijin Zhang

**Affiliations:** Department of Cardiology, The First Hospital of China Medical University, Shenyang, China

**Keywords:** hypertensive nephropathy (HN), weighted gene co-expression network analysis (WGCNA), differentially expressed genes (DEGs), pathogenesis, key gene, *POLR2I*

## Abstract

Hypertensive nephropathy (HN), mainly caused by chronic hypertension, is one of the major causes of end-stage renal disease. However, the pathogenesis of HN remains unclarified, and there is an urgent need for improved treatments. Gene expression profiles for HN and normal tissue were obtained from the Gene Expression Omnibus database. A total of 229 differentially co-expressed genes were identified by weighted gene co-expression network analysis and differential gene expression analysis. These genes were used to construct protein–protein interaction networks to search for hub genes. Following validation in an independent external dataset and in a clinical database, *POLR2I*, one of the hub genes, was identified as a key gene related to the pathogenesis of HN. The expression level of *POLR2I* is upregulated in HN, and the up-regulation of *POLR2I* is positively correlated with renal function in HN. Finally, we verified the protein levels of *POLR2I in vivo* to confirm the accuracy of our analysis. In conclusion, our study identified *POLR2I* as a key gene related to the pathogenesis of HN, providing new insights into the molecular mechanisms underlying HN.

## Introduction

Hypertension is a disease that usually leads to the impairment of target organs, especially kidney. Hypertensive nephropathy (HN), mainly caused by chronic hypertension, is one of the major causes of end-stage renal disease. It has been estimated that 60–90% of patients with chronic kidney disease are hypertensive (Ku et al., 2019). However, existing data have demonstrated that even when blood pressure is reduced to the recommended goal, it can only slow, but not stop, the progression of HN (Udani et al., 2011). Therefore, in addition to effectively reducing blood pressure, it is particularly important to understand the pathogenesis of hypertensive nephropathy. The tubulointerstitial compartment constitutes 95% of the total kidney mass (Berthier et al., 2012), and the tubulointerstitial changes in HN patients are deemed as a major determinant in the development of renal damage (Nath, 1992). Moreover, interstitial changes in hypertension-induced renal injury occurs before glomerular changes become apparent, suggesting that tubulointerstitial compartments may be the crucial initial site of injury (Mai et al., 1993). However, the detailed molecular mechanism of tubulointerstitial lesions in HN is poorly understood.

A growing body of evidence supported that genetic background affects the progression of nephropathy in HN patients (Zhang et al., 2013; Guo et al., 2019; Sun et al., 2020). Weighted gene co-expression network analysis (WGCNA) (Horvath and Dong, 2008) is an effective bioinformatics approach for constructing a co-expression network based on gene expression data profile, which provides new insights for predicting co-expressed genes related to clinical traits and the pathogenesis of diseases. Differential gene expression analysis is a widely used and excellent bioinformatics method to detect changes in gene expression levels between different groups (Segundo-Val and Sanz-Lozano, 2016). Thus, WGCNA and differential gene expression analysis were combined to screen key genes related to the pathogenesis of tubulointerstitial lesions in HN. We hypothesized that the identification of key genes would provide a new insight into HN biomarker discovery.

In the present study, datasets from Gene Expression Omnibus (GEO) were adopted to establish a gene co-expression network and to identify differentially expressed genes (DEGs) between HN tubulointerstitial tissues and matched controls. Then, the overlapping genes that are present in DEGs and the trait-related modules were used to construct a protein–protein interaction (PPI) network to select hub genes. Meanwhile, Gene Ontology (GO) and Kyoto Encyclopedia of Genes and Genomes (KEGG) analysis were performed to assess the potential functions of the overlapping genes. After GEO and clinical validation, a key gene was screened from hub genes. Finally, the key gene was validated by *in vivo* experiments, and the potential biological functions of the key gene was investigated by gene set variation analysis (GSVA).

## Materials and Methods

### Data Collection and Data Pre-processing

**FIGURE 1:**
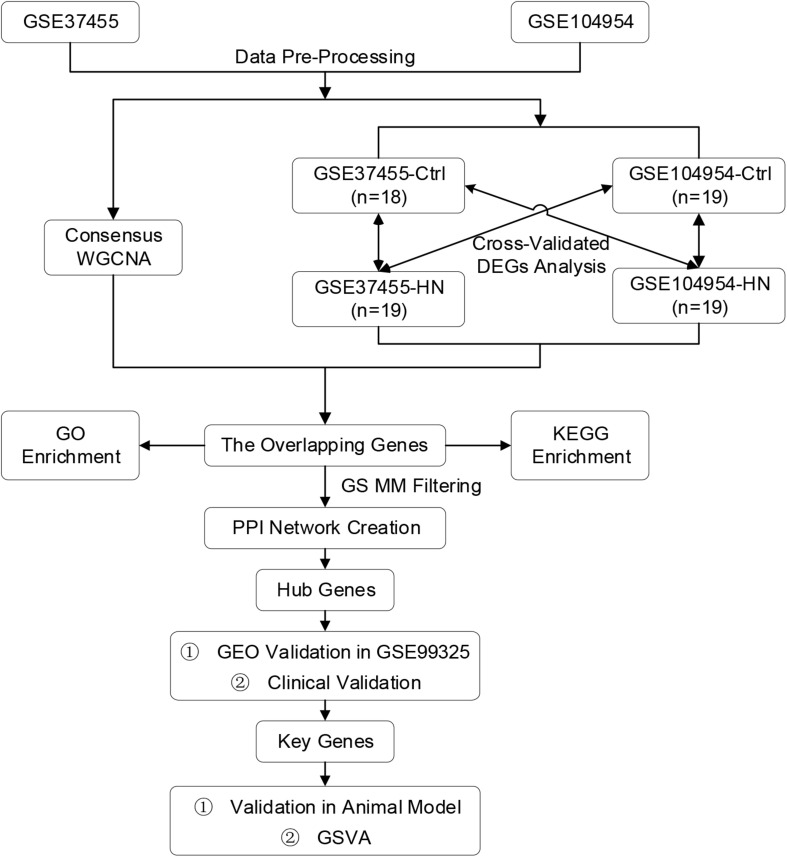
Flowchart of the study including data pre-processing, analysis, and experimental verification. DEGs, differentially expressed genes; GEO, Gene Expression Omnibus; GO, Gene Ontology; GS, gene significance; GSE, Gene Expression Omnibus Series; GSVA, gene set variation analysis; HN, hypertensive nephropathy; KEGG, Kyoto Encyclopedia of Genes and Genomes; MM, module membership; PPI, protein-protein interaction; WGCNA, weighted gene co-expression network analysis.

All HN and healthy control tubulointerstitial tissue samples were selected from the GEO database^1^ with the GSE numbers of GSE37455 (Berthier et al., 2012), GSE104954 (Grayson et al., 2018), and GSE99325 (Shved et al., 2017). These datasets were in accord with the following criteria: (1) containing both HN and healthy control tubulointerstitial tissues, (2) including at least six HN tubulointerstitial samples, (3) the species was *Homo sapiens*, and (4) complete expression profiles were available. The gene expression profiles of these datasets were collected from tubulointerstitial compartments of kidney biopsies from HN patients and healthy control donors (Table 1), whose clinical information is provided in Supplementary Tables 1–3, respectively. More detailed clinical characteristics could be found in the corresponding references (Table 1). Each transcriptome array was normalized independently using Robust Multiarray Average, followed by quantile normalization and log2 transformation. The removal of batch effects was performed by ComBat method, which is a widely used and highly effective method, particularly with smaller sample sizes (Chen et al., 2011). Principal component analysis (PCA) and relative log expression (RLE) analysis were performed to evaluate the removal of batch effect. After using hierarchical clustering to identify and exclude outliers, samples from the HN and control groups were used for a subsequent analysis. The flowchart of our study is shown in Figure 1.

**TABLE 1:** Datasets used in this study.

**GEO dataset**	**Tissue**	**Platform**	**HN**	**Healthy control**	**References**
GSE37455	Tubulointerstitial	GPL11670 (Affymetrix Human Genome U133 Plus 2.0 Array)	0	18	Berthier et al., 2012; Ju et al., 2015
		GPL14663 (Affymetrix GeneChip Human Genome HG-U133A Custom CDF)	20	3	
GSE104954	Tubulointerstitial	GPL22945 [(HG-U133_Plus_2) Affymetrix Human Genome U133 Plus 2.0 Array]	0	18	Ju et al., 2013; Grayson et al., 2018
		GPL24120 [(HG-U133A) Affymetrix Human Genome U133A Array]	20	3	
GSE99325	Tubulointerstitial	GPL19184 [(HG-U133A) Affymetrix Human Genome U133A Array]	20	4	Shved et al., 2017

*GEO, Gene Expression Omnibus; GPL, Gene Expression Omnibus Platform; GSE, Gene Expression Omnibus Series; HN, hypertensive nephropathy.*

### Weighted Gene Co-expression Network Analysis

After filtering out genes with an average expression value of less than 1, the top 75% of genes with the largest median absolute deviation in the samples were selected to construct a co-expression network using the R package “WGCNA” (Langfelder and Horvath, 2008). When 0.85 was used as the correlation coefficient threshold, the most appropriate soft-thresholding powers (β) value with the maximum average connectivity was selected from 1 to 20. Then, the scale-free network was transformed into a topological overlap matrix (TOM) according to the most proper β. Based on the TOM, co-expression modules were defined as branches of a hierarchical cluster tree using the dynamic tree cut method with a minimum size cutoff of 50. To merge possible similar modules, we defined 0.2 as the threshold for cut height. Furthermore, the adjacency of module eigengenes (Horvath and Dong, 2008) and the correlation among randomly picked genes were calculated to evaluate the reliability of the constructed modules. The results were visualized using heat maps. Next, the trait-related modules that were highly correlated with clinical traits were selected by us for a subsequent analysis. The gene significance (GS) and module membership (MM) of trait-related modules were calculated and visualized using a scatter plot diagram.

### Differential Gene Expression Analysis and Identification of Overlapping Genes

The DEGs between the HN and control samples were defined using the “limma” R package (Ritchie et al., 2015). False discovery rate (FDR) adjusted *p*-values < 0.05 and | log FC| > 0.5 in all four comparisons were considered statistically significant. The PCA of DEGs was applied to show the clustering of HN and control samples, and then the overlapping genes that are present in trait-related modules and DEGs were analyzed to identify potential hub genes. The differential expression of the overlapping genes was visualized using a heat map.

### Function Enrichment Analyses

To further explore the biological significance of the overlapping genes, we used the R package “clusterprofiler” (Yu et al., 2012) to conduct GO function and KEGG pathway enrichment analyses. The cutoff value was set to *p*-value < 0.05.

### PPI Network Creation and Identification of Hub Genes

In order to identify real hub genes, we first screened genes from the overlapping genes according to the criteria that the GS and MM of genes were greater than 0.8. Subsequently, the STRING database (Szklarczyk et al., 2021) was used to explore the interactions of the selected genes and to map the PPI network with an integrated confidence score of 0.400. The exported data were visualized in Cytoscape v 3.8 (Kohl et al., 2011), and the CytoHubba plugin (Chin et al., 2014) of Cytoscape was adopted to identify hub genes in the PPI network using degree analysis methods.

### GEO Validation and Clinical Validation

The hub genes were validated in an independent dataset GSE99325. Nephroseq v5 online database,^2^ an integrated bioinformatics platform for mining gene expression datasets for kidney disease, was used to validate the correlation between hub genes and the clinical traits of HN. A correlation analysis was performed by applying Pearson’s method, and *P* < 0.05 was considered as statistically significant.

### Animal Model of AngII-Induced Hypertension

Wild-type (WT) male mice (8–10 weeks old) were purchased from the Shanghai Biomodel Organism Science and Technology Development. Hypertensive renal injury was induced by NaCl (*n* = 6) or angiotensin II (AngII) (*n* = 6) (A9525, Sigma, United States; 1.5 mg kg^–1^ day^–1^), respectively, for 28 days *via* Alzet minipumps (Alzet, model 2002; 0.5 μl/h) as described previously (Jia et al., 2021). Blood pressure measurement was carried out by the tail–cuff method. Kidney tissue samples were collected at day 28 for histology and immunofluorescence. All the mice were raised and handled in strict accordance with the animal welfare regulations of China Medical University and in compliance with the National Institutes of Health Guide for the Care and Use of Laboratory Animals.

### Western Blotting

The detailed protocol for western blotting has been previously described (Zhang et al., 2020). Briefly, after transferring the kidney total protein to the poly-vinylidene difluoride membrane, the membrane was incubated overnight with primary antibodies. Followed by the corresponding secondary antibodies for 1 h at room temperature, signals were captured and visualized using enhanced chemiluminescence. The ratio of the protein of interest was subjected to α-tubulin and was analyzed by ImageJ version 1.46 (National Institutes of Health, United States). The following antibodies were used: anti-POLR2I (1:500; Santa Cruz Biotechnology, United States), anti-collagen I (1:500; Proteintech, United States), anti-α-SMA (1:1,000; Proteintech, United States), anti-α-tubulin (1:1,000; Proteintech, United States).

### PAS Staining, MT Staining, and Immunofluorescence Staining

Periodic acid–Schiff (PAS) staining was performed to assess the glomerular damage of the kidney, and Masson’s trichrome (MT) staining was performed to assess the fibrosis of the kidney. As for immunofluorescence staining, an anti-POLR2I (1:50) and the donkey anti-mouse IgG (H + L) Highly Cross-Adsorbed Secondary Antibody, Alexa Fluor 488 (Invitrogen, 1:200) were used to fluorescently label POLR2I in the tubulointerstitium of the kidney. Digital images were then scanned and captured with a fluorescence microscope (Nikon Eclipse 90i, Japan).

### GSVA

The HN samples from GSE37455, GSE104954, and GSE99325 were, respectively, divided into two groups (high expression *vs*. low expression) based on the median expression value of the key gene. We utilized the R package “GSVA” to find pathways and functions most relevant to the key gene (Hänzelmann et al., 2013) according to the gene set files from the Molecular Signature Database (MSigDB^3^). *P* < 0.05 in all datasets was considered statistically significant.

### Statistical Analysis

GraphPad Prism version 8 (GraphPad Software, La Jolla, CA) was used for data analysis. All values are expressed as mean ± SEM and were analyzed with the Student’s *t*-test. *P*-value < 0.05 was considered statistically significant.

## Results

### Data Collection and Data Pre-processing

**FIGURE 2:**
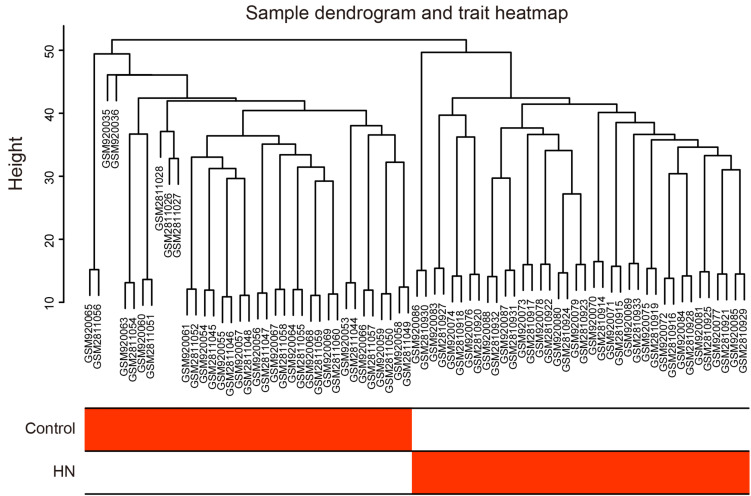
Hierarchical clustering dendrogram and trait heat map. The branches of the dendrogram correspond to clustered samples. HN, hypertensive nephropathy.

A total of 106 tubulointerstitial tissue samples including 60 HN cases and 46 healthy control subjects were obtained from GSE37455, GSE104954, and GSE99325 datasets (Table 1). In our study, GSE37455 and GSE104954 were analyzed to find hub genes related to the tubulointerstitial lesions in HN, and GSE99325 was used to verify the external stability of hub genes. Notably, GSE37455 was processed by two platforms, GPL11670 and GPL14663; GSE104954 was also processed by two platforms, GPL22945 and GPL24120. Therefore, we used the ComBat method to eliminate the batch effects. The scatter plots based on PCA and the boxplots based on RLE analysis (Supplementary Figures 1A–D) revealed that the batch effects from different platforms were successfully removed. Subsequently, five outliers in the control group and two outliers in the HN group were detected and excluded (Supplementary Figures 1E,F) by hierarchical clustering analysis. As shown in Figure 2, the remaining samples were divided into two clusters, indicating a high level of consistency between samples of the same type.

### Construction of the WGCNA Network and Selection of Trait-Related Modules

**FIGURE 3:**
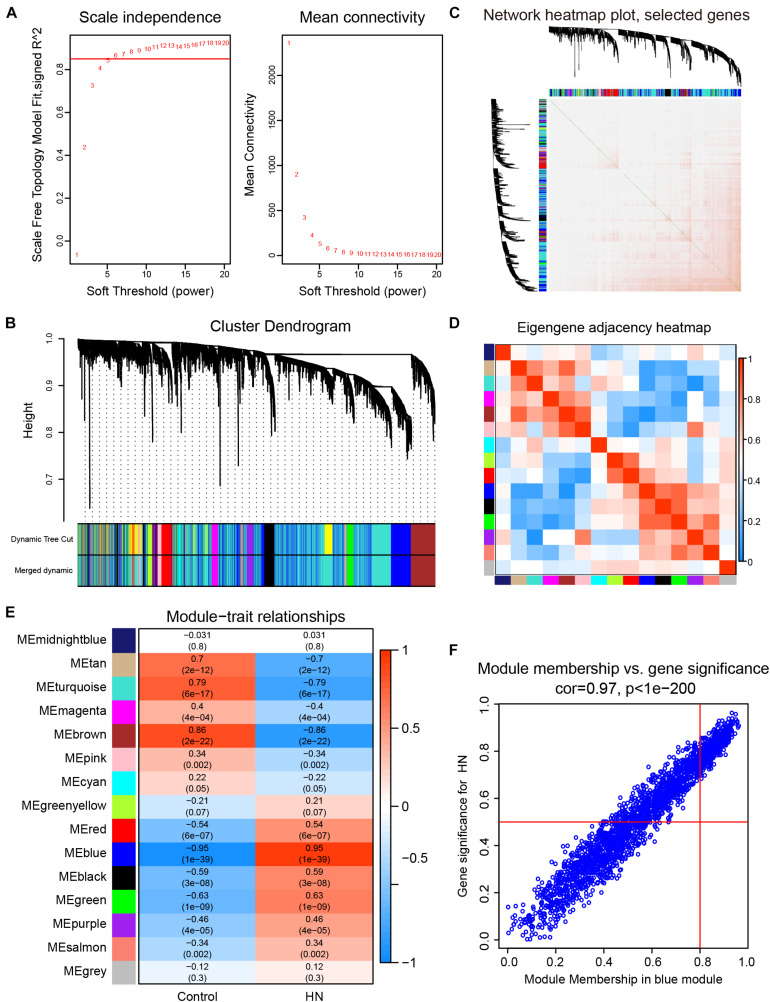
Construction of the weighted gene co-expression network and selection of trait-related modules. **(A)** Analysis of the scale-free fit index (left) and the mean connectivity (right) for various soft-thresholding powers. **(B)** Cluster dendrogram. The original and merged modules are shown in the two colored bars below the dendrogram obtained by the dynamic tree cut method. **(C)** Heat map of TOM of 1,000 selected genes. The colors from light to dark red represent a low to high topological overlap. The gene dendrogram and module allocation are shown along the left and top. **(D)** Eigengene adjacency heat map. The colors of the squares from blue to red indicate the adjacency of the corresponding modules from low to high. **(E)** Module–trait relationships heat map. The *R*-value for each correlation and *P*-value in parenthesis are shown in the cells. **(F)** Scatter plot of gene significance for HN related to module membership in blue module. HN, hypertensive nephropathy; ME, module eigengene; TOM, topological overlap matrix.

We selected β = 6 as the soft-thresholding power to construct a scale-free network (Figure 3A). The gene co-expression modules were identified using a merged dynamic tree cut and represented by different colors (Figure 3B). The TOM heat map of 1,000 randomly selected genes revealed that each module was independent of the others (Figure 3C). In addition, most modules had a low adjacency to the others, revealing that the clustering was independent and accurate (Figure 3D).

A correlation heat map between modules and traits is shown in Figure 3E. The blue module (2,198 genes) was the most positively associated with HN (*R* = 0.95, *P* = 1e-39), while the brown module (696 genes) was the most positively related to the control trait (*R* = 0.86, *P* = 2e-22). In order to ensure the stability and the reliability of the results, we selected the blue module that is most relevant to the traits for further analysis. Moreover, the WGCNA analysis between each control subgroup and each HN subgroup also showed that the blue module had the highest correlation with the traits (Supplementary Figures 2A–D), indicating the high stability of our results. In the blue module, scatterplots of the GS *vs.* MM were plotted (Figure 3F), in which the GS and MM had a high correlation [cor = 0.97 (*p* < 1e-200)]. These results suggested that the blue module was the most valuable module for finding hub genes related to HN.

### Identification of DEGs and Overlapping Genes

**FIGURE 4:**
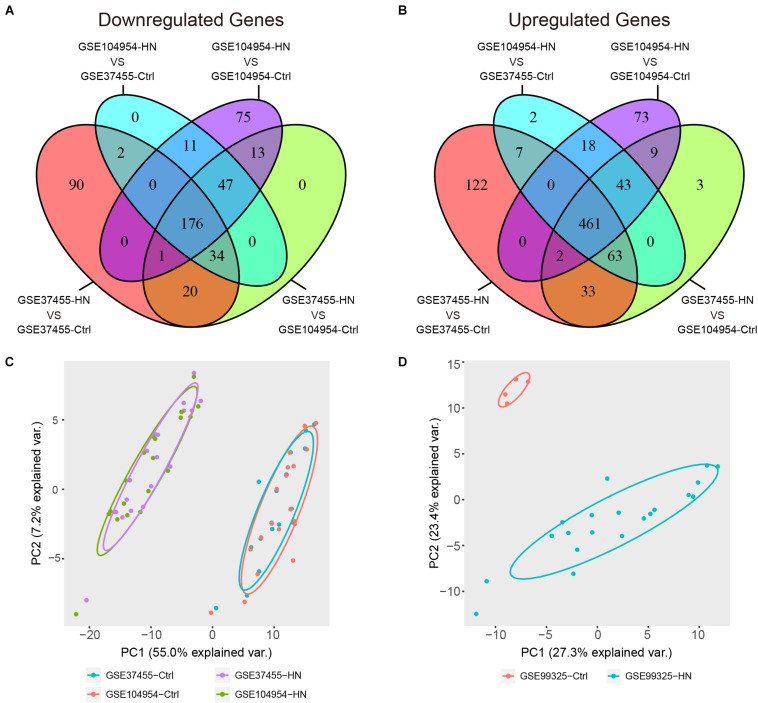
Identification of DEGs. **(A,B)** Venn diagram showing a total of 637 DEGs identified by cross-validated comparisons, including 176 downregulated genes **(A)** and 461 upregulated genes **(B)**. The screening criteria for DEGs was set as | log FC| > 1 and FDR (adjusted *P*-value) < 0.05 in all four comparisons. **(C)** Principal component plots of the 637 DEGs showing an overlap between samples from each of the HN or control groups and separation between disease and control in the expression profiles after batch-effect removal. **(D)** Principal component plot of the 637 DEGs showing a separation between samples from the HN group or control group in independent external dataset GSE99325. DEGs, differentially expressed genes; FC, fold change; FDR, false discovery rate; GSE, Gene Expression Omnibus Series; HN, hypertensive nephropathy; ME, module eigengene.

By a cross-validated comparison of HN and control groups, we detected 637 DEGs, including 176 genes that were downregulated and 461 genes that were upregulated in HN (Figures 4A,B). These results indicated that most genes were abnormally upregulated in HN compared with normal controls. The PCA of the DEGs showed an overlap of samples within the HN or control groups and a good separation between HN and control (Figure 4C). The DEGs were also used to plot the separation between the HN group and the control group samples in an independent dataset, GSE99325 (Figure 4D).

**FIGURE 5:**
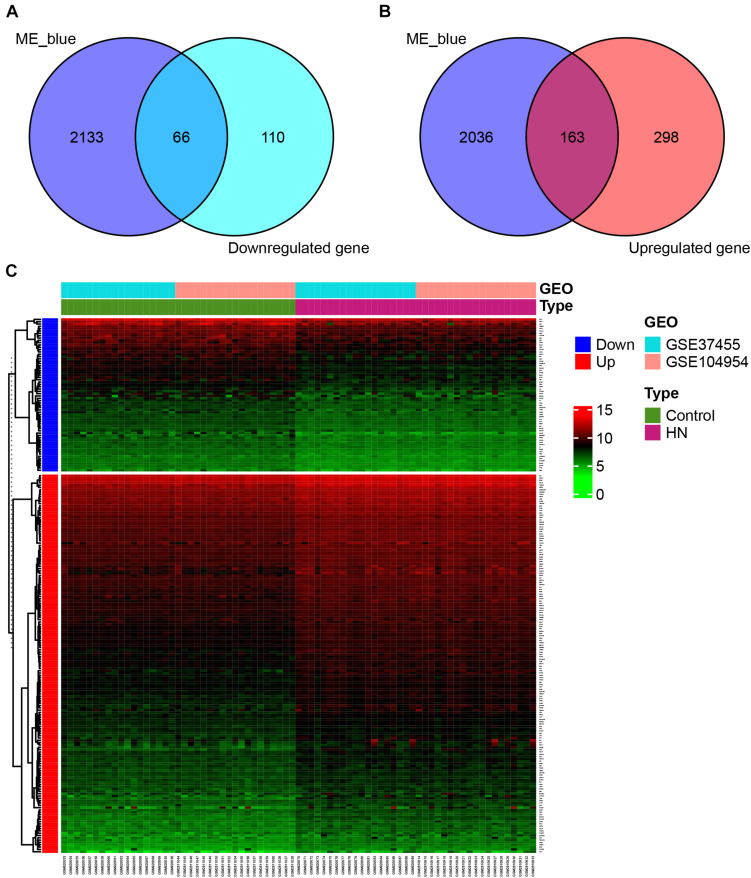
Identification of overlapping genes. **(A,B)** Selection of overlapping genes. Venn diagram showing 66 downregulated **(A)** and 163 upregulated **(B)** overlapping genes present in DEGs and the genes in the blue module. **(C)** Heat map of gene expression for 229 overlapping genes. GEO, Gene Expression Omnibus; GSE, Gene Expression Omnibus Series; HN, hypertensive nephropathy; ME, module eigengene.

We detected a total of 229 overlapping genes that are present in the blue module and DEGs, including 66 downregulated overlapping genes (Figure 5A) and 163 upregulated overlapping genes (Figure 5B). The overlapping genes were deemed as potential hub genes related to HN. A gene expression heat map for the 229 overlapping genes is shown in Figure 5C.

### Enrichment Analyses of Overlapping Genes

**FIGURE 6:**
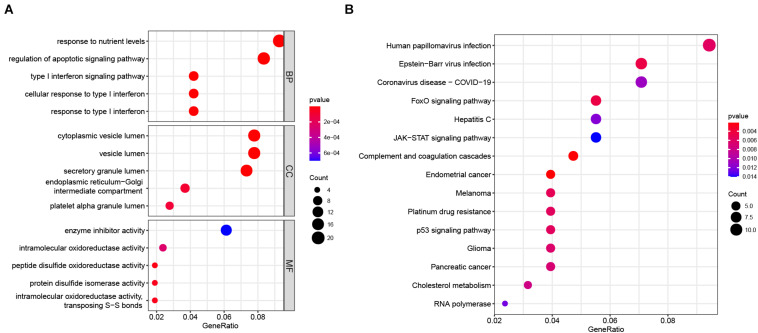
GO and KEGG enrichment analysis of 229 overlapping genes. **(A)** GO enrichment analysis. The GO enrichment analysis consisted of three main panels, namely, BP, CC, and MF. **(B)** KEGG pathway enrichment analysis. BP, biological process; CC, cellular component; GO, Gene Ontology; KEGG, Kyoto Encyclopedia of Genes and Genomes; MF, molecular function.

We performed GO and KEGG enrichment analyses on the 229 overlapping genes. The biological process (BP) of the overlapping genes was mainly related to immune response, such as “type I interferon signaling pathway”, “cellular response to type I interferon”, “response to type I interferon”, and so on (Figure 6A). In terms of cellular component (CC), the enriched genes were primarily associated with “cytoplasmic vesicle lumen”, “vesicle lumen”, “secretory granule lumen”, and so on (Figure 6A). As to molecular function (MF), the enriched genes were primarily associated with redox activity, such as “intramolecular oxidoreductase activity”, “peptide disulfide oxidoreductase activity”, “protein disulfide isomerase activity”, “intramolecular oxidoreductase activity, transposing S-S bonds”, and so on (Figure 6A). Regarding the KEGG pathway analysis, the enriched genes mostly related to “human papillomavirus infection” and so on (Figure 6B). In addition, most genes were also enriched in “RNA polymerase” (Figure 6B).

### PPI Network Creation and Identification of Hub Genes

**FIGURE 7:**
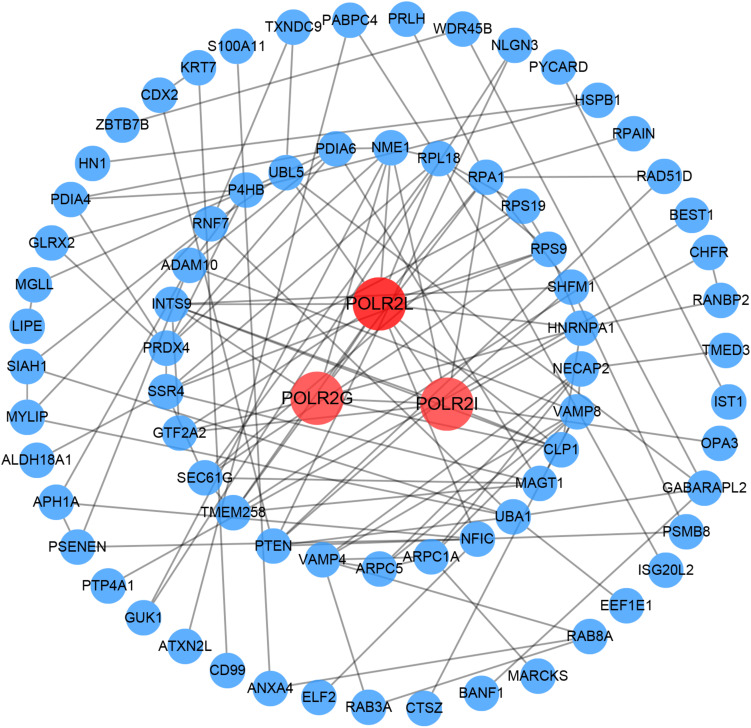
PPI network creation. A PPI network with 75 nodes and 131 edges was created in the 116 overlapping genes whose GS and MM were both greater than 0.8. The genes with the top three degree values are shown in red and orange in the PPI network. The darker the color, the greater the degree value representing connectivity with other genes in the PPI network. GS, gene significance; MM, module membership; PPI, protein–protein interaction.

Based on GS > 0.8 and MM > 0.8, 116 genes (Supplementary Table 4) were extracted from the overlapping genes to construct PPI network. Then, we visualized the PPI network using Cytoscape and calculated the degree values of each gene using the Cytohubba plug-in. Finally, *POLR2L*, *POLR2G*, and *POLR2I* were identified as hub genes according to the degree values ranking, which have the highest connectivity in the PPI network (Figure 7).

### GEO Validation and Clinical Validation

**FIGURE 8:**
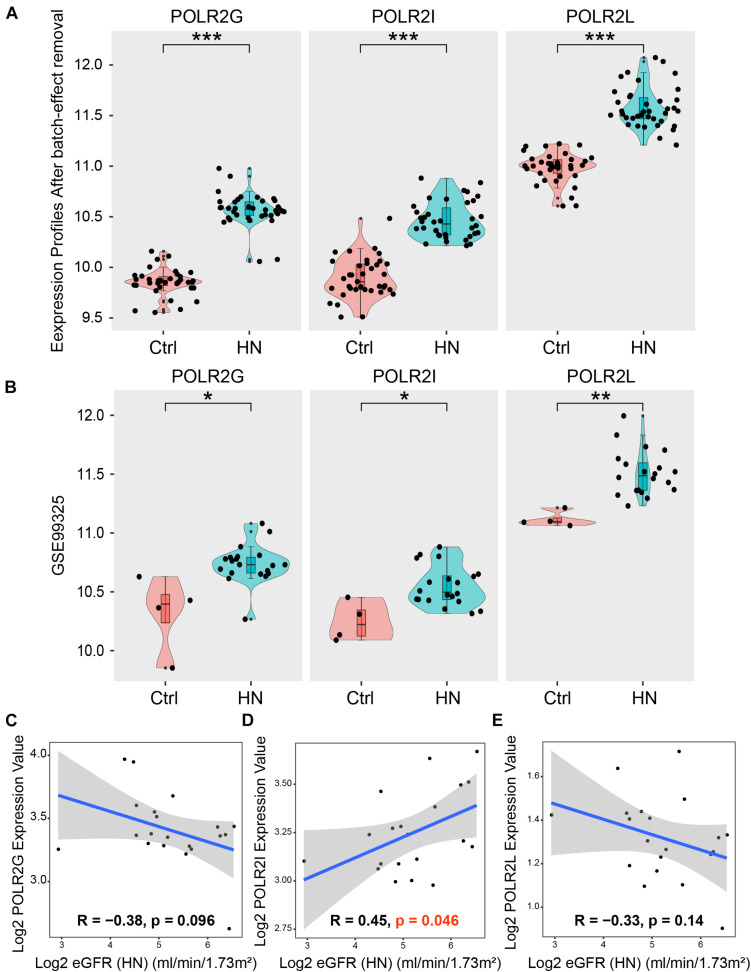
GEO validation and clinical validation. **(A)** The expression of hub genes in the expression profiles after batch-effect removal. **(B)** GEO validation. The expression of hub genes in an independent external dataset GSE99325. ***p < 0.001; **p < 0.01; *p < 0.05. **(C–E)** Clinical validation. Correlation between the expression of hub genes in HN tubulointerstitium and eGFR. eGFR, estimated glomerular filtration rate; GEO, Gene Expression Omnibus; GSE, Gene Expression Omnibus Series; HN, hypertensive nephropathy.

We analyzed the expression of hub genes in the expression profiles after batch-effect removal and found that hub genes were overexpressed in HN compared with the healthy controls (Figure 8A). These results were consistent with the analysis in an independent external dataset GSE99325, thus verifying our findings (Figure 8B). According to the National Kidney Foundation Kidney Disease Outcomes Quality Initiative (NKF KDOQI) Clinical Practice Guidelines, the estimated glomerular filtration rate (eGFR) is the best overall index of kidney function in health and disease (Levey et al., 2014). It is considered to reflect the pathological process of the kidney and is therefore widely used as the primary outcome measure in most studies (Lambers Heerspink et al., 2014; Ju et al., 2015). Hence, the Nephroseq v5 online database was used to explore the correlation between the expression values of hub genes and eGFR in the HN. As shown in Figures 8C–E, among hub genes, only the expression of *POLR2I* was significantly positively correlated with eGFR (*R* = 0.45, *p* < 0.05) in HN, thus indicating the important role of *POLR2I* gene in HN. The above-mentioned results revealed that *POLR2I* is a key gene associated with HN, and the up-regulation of *POLR2I* is positively correlated with renal function in HN, which may be related to the progression of HN.

### *In vivo* Validation of the Key Gene

**FIGURE 9:**
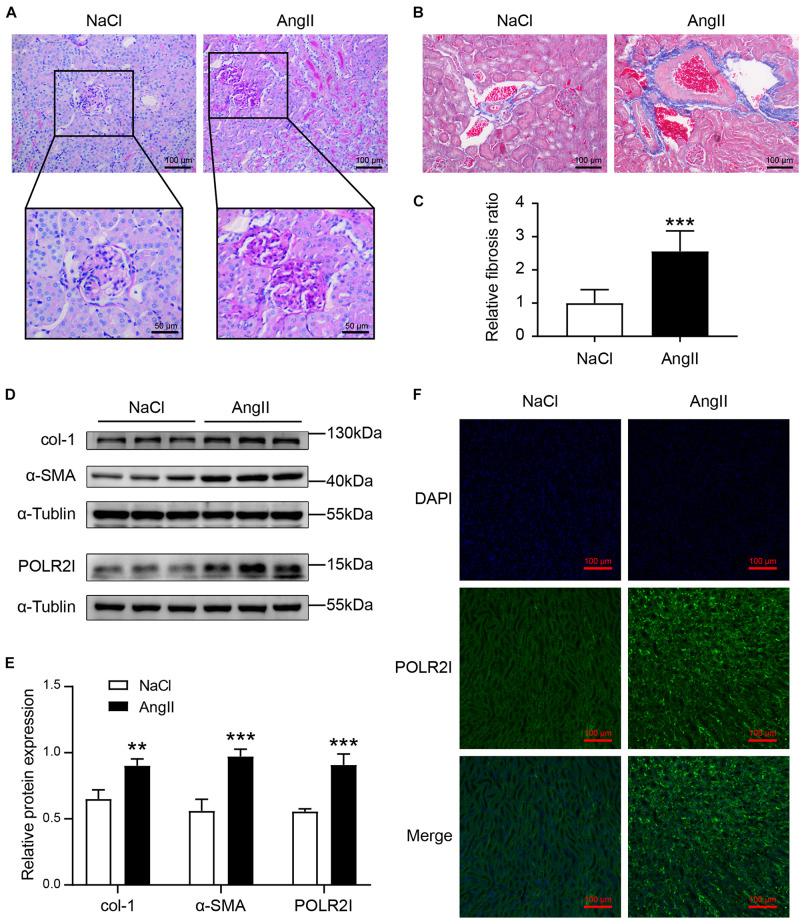
The expression of POLR2I protein was upregulated in tubulointerstitium of AngII-treated mice relative to NaCl-treated mice. **(A)** Representative microscopy images of PAS staining of kidney sections from the NaCl group and AngII group. The lower panel showed the zoomed-in images of glomerulus of the NaCl group and AngII group (upper original magnification, ×200; scale bar = 100 μm; lower original magnification, ×400; scale bar = 50 μm). **(B,C)** Representative microscopy images and quantification of MT staining of kidney sections from the NaCl group and AngII group (original magnification, ×200; scale bar = 100 μm). ***P < 0.001, *n* = 6. **(D,E)** Representative Western blots and quantification of col-1, α-SMA, and POLR2I protein levels in the NaCl group and AngII group. ***P < 0.001, **P < 0.01, *n* = 3. **(F)** Representative microscopy images of POLR2I immunofluorescence staining of kidney sections from the NaCl group and AngII group (original magnification, ×200; scale bar = 100 μm) (POLR2I green, DAPI blue). AngII, angiotensin II; MT, Masson’s trichrome; PAS, periodic acid-Schiff.

AngII is a key mediator of hypertension and hypertension-associated organ damage, which can induce the pathological characteristics of HN (Lu et al., 2019). As shown in Figure 9A, chronic AngII infusion resulted in significant glomerular abnormalities, characterized by disordered glomerular clusters, mesangial hyperplasia, and mesangial matrix expansion. Similarly, chronic AngII infusion resulted in significant renal fibrosis, as evidenced by MT staining (Figures 9B,C) and the expression of fibrosis makers col-1 and α-SMA (Figures 9D,E). These results demonstrated that we successfully constructed an animal model of hypertensive renal injury. Western blotting detected that the expression of the *POLR2I* protein was significantly upregulated in AngII-treated mice compared with the NaCl-treated mice (Figures 9D,E). In addition, immunofluorescence staining detected that the expression of the *POLR2I* protein was significantly upregulated in the tubulointerstitium of AngII-treated mice relative to NaCl-treated mice (Figure 9F). These findings suggested that *POLR2I* is overexpressed in the tubulointerstitium of HN and may be involved in the tubulointerstitium lesions of HN, which confirmed the results of our analysis.

### GSVA

**FIGURE 10:**
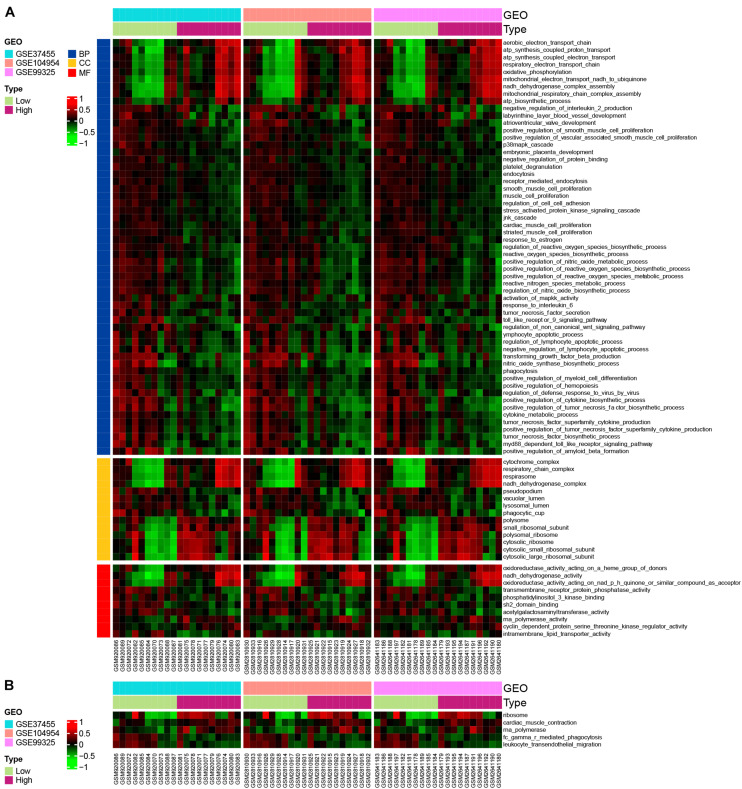
GSVA of *POLR2I*. GSVA was used to explore biological function **(A)** and KEGG pathways **(B)** between low- and high-*POLR2I*-expression groups in HN. Only GO terms or KEGG pathways with *P* < 0.05 in all datasets are shown. BP, biological process; CC, cellular component; GEO, gene expression omnibus; GO, Gene Ontology; GSE, Gene Expression Omnibus Series; GSVA, gene set variation analysis; HN, hypertensive nephropathy; KEGG, Kyoto Encyclopedia of Genes and Genomes; MF, molecular function.

In order to explore the most relevant pathways and biological processes with *POLR2I* gene, we performed GSVA on the HN samples in GSE37455, GSE104954, and GSE99325. In terms of BP, many gene sets associated with oxidative stress, such as “positive regulation of reactive oxygen species metabolic process”, “positive regulation of reactive oxygen species biosynthetic process”, and “positive regulation of nitric oxide metabolic process”, were downregulated in the *POLR2I* high-expression groups (Figure 10A). In terms of CC, gene sets associated with ribosomes, such as “small ribosomal subunit”, “cytosolic small ribosomal subunit”, and “cytosolic ribosome”, were upregulated in the *POLR2I* high-expression groups (Figure 10A). In terms of MF, the gene set “RNA polymerase activity” and many other gene sets associated with redox activity, such as “oxidoreductase activity acting on a heme group of donors” and “NADH dehydrogenase activity”, were upregulated in the *POLR2I* high-expression groups (Figure 10A). Meanwhile, Figure 10B shows that the “RNA polymerase” and “ribosome” pathways were significantly upregulated in the high-expression groups of *POLR2I*. More detailed results of GSVA analysis are available in Supplementary Tables 5,6.

## Discussion

In this study, we utilized integrated bioinformatics to analyze the gene expression profiles in HN and healthy controls from two independent GEO datasets. A total of 229 critical genes with co-expression trends were identified in the datasets. The GO and KEGG enrichment analyses showed that these genes primarily participated in various processes associated with transcription activity and redox activity. These results indicated that HN is possibly a disorder caused by abnormalities in transcription activity and imbalance in redox homeostasis. As previously reported, the abnormal transcription activity of genes (Zhang et al., 2015; Dhande et al., 2020) and oxidative stress (Majzunova et al., 2019; Nguyen et al., 2020) can be a cause or, more often, a potentiating factor for hypertension and hypertensive renal disease. Besides this, three hub genes associated with HN, namely, *POLR2L*, *POLR2G*, and *POLR2I*, were identified by PPI network analysis. Gene *POLR2L*, encoding a subunit of RNA polymerase II, may be at the apex of a post-transcriptional regulatory cascade, promoting the bloodstream-form trypanosome differentiation state (Mugo and Clayton, 2017). Gene *POLR2G* encodes the seventh largest subunit of RNA polymerase II, which is involved in a wide range of gene expression regulation, including transcription, mRNA export and decay, and translation (Allepuz-Fuster et al., 2014). Gene *POLR2I* is located on chromosome 19q13.12 and encodes a small core subunit of RNA polymerase II, which is involved in DNA repair and multiple transcription-related processes (Iben et al., 2011; Berkyurek et al., 2021).

After validation in an independent GEO dataset and a clinical database, *POLR2I*, one of the hub genes, was identified as a key gene involved in the development of HN. We found that *POLR2I* is overexpressed in HN and that the upregulation of *POLR2I* is positively correlated with renal function in HN, which may be important in the progress of HN. Finally, the results of our analysis were verified by an *in vivo* experiment. The maintenance of transcriptional fidelity and genome integrity is essential for cellular functions, while deregulation of transcription and defects in DNA repair can cause serious pathologies, including HN (Trudu et al., 2013; Georges et al., 2019). It was reported that the absence of POLR2I significantly reduces transcriptional accuracy (Nesser et al., 2006) and also leads to defects in replication fork progression (Felipe-Abrio et al., 2015). Moreover, POLR2I mediates transcription-coupled repair (TCR) pathways (Li and Smerdon, 2002), and the loss of POLR2I can lead to a defective DNA damage response (Sein et al., 2018). Therefore, POLR2I, as the main component of the transcription and DNA repair machineries (Georges et al., 2019), may play a key role in the pathological process of HN.

Currently, the relationship between *POLR2I* and HN has not been previously reported. The GSVA analysis of *POLR2I* showed that many gene sets associated with oxidative stress were downregulated in the *POLR2I* high-expression groups (Figure 10A), suggesting the potential functions of *POLR2I* in renal oxidative stress damage associated with HN. Consistent with our enrichment analysis results (Figure 6A), there is evidence that oxidative stress is an important driving factor of the pathogenesis and progression of HN (Wilcox, 2005; Balhorn et al., 2020). Oxidative stress can induce DNA damage, which can be fatal to cells if not repaired (You et al., 2020). Notably, *POLR2I* plays a pivotal role in TCR pathways and exhibits a genetic interaction with genes in various DNA repair pathways (Chen et al., 2007; Georges et al., 2019). Hence, we hypothesized that *POLR2I* is possibly involved in HN by affecting various processes associated with oxidative stress. However, this hypothesis has not been reported before, and more work is needed to confirm this hypothesis.

The focus of our study was to investigate the molecular mechanisms of tubulointerstitial lesions in the HN rather than glomerular lesions (Cui et al., 2017; Liu et al., 2018). It is based on the hypothesis that subtle tubulointerstitial lesions are not merely the manifestation of HN but an underlying cause of hypertension (Johnson et al., 2002). A previous study (Chen et al., 2019) adopted differential gene expression analysis to discover key genes in the tubulointerstitial lesions of HN. However, this method of simply using DEGs analysis can ignore the highly correlated links between genes and may filter out genes that have a high interconnectivity in the network. Therefore, our study combined the WGCNA method and the DEGs method to search for key genes associated with HN. In addition, our findings were also considered credible and stable after GEO validation in an independent dataset and clinical validation in the Nephroseq v5 online database. Finally, the accuracy of our analysis was verified by an *in vivo* experiment.

One limitation of the study was that, while we performed the Combat method, we could not completely eliminate the batch effects in the GSE37455 and GSE104954 datasets, in which hub genes were identified. We rather believed that validation in an independent GEO dataset and in a clinical database, as well as further validation *in vivo*, provided sufficient robust validation of the findings. While our study identified and validated the key gene for tubulointerstitial lesions in HN using datasets with a larger sample size, these studies were also conducted in quite distinct populations. Thus, the role of the key gene still needs to be confirmed in more different races.

In summary, we identified *POLR2I* as a key gene related to HN by comprehensive bioinformatics analysis. Our study provides a new insight into the molecular mechanisms underlying HN and offers a new candidate target for the precise treatment of the disease, although more work is needed to fully reveal the role of *POLR2I* in the pathogenesis of HN.

## Data Availability Statement

Gene expression profiling datasets for this study can be found in Gene Expression Omnibus (GEO, http://www.ncbi.nlm.nih.gov/geo/), reference number GSE37455, GSE104954, and GSE99325. The clinical information was derived from the following resources available in the public domain: Nephroseq v5 online database (http://v5.nephroseq.org). And the original contributions presented in the study are included in the article/Supplementary Material.

## Ethics Statement

The animal study was reviewed and approved by Animal Care and Use Committee of China Medical University.

## Author Contributions

YS, NZ, and YZ designed the study. BW and SW collected research data. SY, JX, BW, and SW performed data analysis. SY and JX wrote the manuscript. NZ revised the manuscript. All authors read and approved the final manuscript.

## Conflict of Interest

The authors declare that the research was conducted in the absence of any commercial or financial relationships that could be construed as a potential conflict of interest.

## Publisher’s Note

All claims expressed in this article are solely those of the authors and do not necessarily represent those of their affiliated organizations, or those of the publisher, the editors and the reviewers. Any product that may be evaluated in this article, or claim that may be made by its manufacturer, is not guaranteed or endorsed by the publisher.

## Footnotes

1
http://www.ncbi.nlm.nih.gov/geo/

2
http://www.v5.nephroseq.org

3
http://software.broadinstitute.org/gsea/msigdb/index.jsp
