# Supplementary material for: Comprehensive Bioinformatics Analysis Identifies POLR2I as a Key Gene in the Pathogenesis of Hypertensive Nephropathy
Source: Front Genet. 2021 Aug 5;12:698570. doi: 10.3389/fgene.2021.698570 (PMC8375388; doi:10.3389/fgene.2021.698570)
Supplement: Supplementary file 1 [file Data_Sheet_1.docx]

Supplementary Material

# Supplementary Figures and Tables

## Supplementary Table 1

|  | Healthy controls | HN |
| --- | --- | --- |
| Number subjects | 15 | 20 |
| Age(years) | 56.9±4.7 | 57.2±12.1 |
| Gender (% female) | 40 | 25 |
| eGFR (ml/min per 1.73 m^2^) | 78.4±6.1 | 43.9±25.1 |

**Supplementary Table 1.** Clinical and demographic information of subjects in the GSE37455 dataset. Age and eGFR are presented as means ± SD. eGFR, estimated glomerular filtration rate; GSE, Gene Expression Omnibus Series; HN, hypertensive nephropathy; SD, standard deviation.

## Supplementary Table 2

|  | Healthy controls | HN |
| --- | --- | --- |
| Number subjects | 21 | 15 |
| Age(years) | 47.3±11.5 | 57.1±11.8 |
| Gender (% female) | 45 | 20 |
| eGFR (ml/min per 1.73 m^2^) | 104±31 | 40.9±23.8 |

**Supplementary Table 2.** Clinical and demographic information of subjects in the GSE104954 dataset. Age and eGFR are presented as means ± SD. eGFR, estimated glomerular filtration rate; GSE, Gene Expression Omnibus Series; HN, hypertensive nephropathy; SD, standard deviation.

## Supplementary Table 3

|  | Healthy controls | HN |
| --- | --- | --- |
| Number subjects | 4 | 20 |
| Age(years) | 54 (50-54) | 57 (40-79) |
| Gender (% female) | 20 | 25 |
| eGFR (ml/min per 1.73 m^2^) | 89.1 (64.9-99.2) | 44.1 (6.4-95.1) |
| Creatinine (mg/dl) | 0.93 (0.90-1.20) | 2.22 (0.73-7.46) |
| Proteinuria (g/24h) | 0.24 (0.03 - 0.45) | 1.38 (0.10-4.90) |

**Supplementary Table 3.** Clinical and demographic information of subjects in the GSE99325 dataset. eGFR, estimated glomerular filtration rate; GSE, Gene Expression Omnibus Series; HN, hypertensive nephropathy; SD, standard deviation.

## Supplementary Table 4

| **Gene Symbol** | **Down or Up** | **GS.HN** | **p.GS.HN*** | **MMblue** | **p.MMblue*** |
| --- | --- | --- | --- | --- | --- |
| NLGN3 | Down | -0.91063 | 9.78E-30 | -0.87216 | 2.29E-24 |
| PPIG | Down | -0.92384 | 3.61E-32 | -0.93653 | 5.83E-35 |
| RANBP2 | Down | -0.87382 | 1.47E-24 | -0.8737 | 1.51E-24 |
| CDX2 | Down | -0.88309 | 1.07E-25 | -0.88319 | 1.04E-25 |
| RAX | Down | -0.86689 | 9.08E-24 | -0.88315 | 1.05E-25 |
| MEF2D | Down | -0.87311 | 1.77E-24 | -0.8611 | 3.86E-23 |
| MAPK8IP2 | Down | -0.87305 | 1.81E-24 | -0.88096 | 2.00E-25 |
| PYY2 | Down | -0.87392 | 1.43E-24 | -0.86502 | 1.46E-23 |
| PRB1 | Down | -0.85596 | 1.32E-22 | -0.88666 | 3.69E-26 |
| OPA3 | Down | -0.88798 | 2.47E-26 | -0.85641 | 1.19E-22 |
| HOXC8 | Down | -0.85545 | 1.49E-22 | -0.81786 | 3.41E-19 |
| HSF4 | Down | -0.87428 | 1.30E-24 | -0.91506 | 1.66E-30 |
| RAB3A | Down | -0.86989 | 4.18E-24 | -0.87354 | 1.58E-24 |
| TACC1 | Down | -0.86427 | 1.76E-23 | -0.91524 | 1.53E-30 |
| PTP4A1 | Down | -0.87885 | 3.64E-25 | -0.85469 | 1.78E-22 |
| ZBTB7B | Down | -0.84784 | 8.42E-22 | -0.88507 | 5.96E-26 |
| BEST1 | Down | -0.86695 | 8.95E-24 | -0.90897 | 1.86E-29 |
| NFIC | Down | -0.85126 | 3.92E-22 | -0.83136 | 2.64E-20 |
| PRLH | Down | -0.84509 | 1.54E-21 | -0.88961 | 1.49E-26 |
| LIPE | Down | -0.85427 | 1.96E-22 | -0.86711 | 8.58E-24 |
| ATXN2L | Down | -0.83842 | 6.33E-21 | -0.85555 | 1.46E-22 |
| VAMP4 | Down | -0.84281 | 2.52E-21 | -0.89811 | 9.32E-28 |
| F7 | Down | -0.81085 | 1.18E-18 | -0.85692 | 1.06E-22 |
| GABARAPL2 | Up | 0.962483 | 4.28E-43 | 0.932266 | 5.81E-34 |
| RAD51D | Up | 0.928446 | 4.02E-33 | 0.878695 | 3.81E-25 |
| CITED2 | Up | 0.9293 | 2.63E-33 | 0.892635 | 5.70E-27 |
| WDR45B | Up | 0.957067 | 5.34E-41 | 0.948393 | 3.79E-38 |
| RPAIN | Up | 0.938316 | 2.13E-35 | 0.919004 | 3.13E-31 |
| ANXA4 | Up | 0.890808 | 1.02E-26 | 0.912926 | 3.94E-30 |
| UBL5 | Up | 0.92591 | 1.37E-32 | 0.930803 | 1.24E-33 |
| SEM1 | Up | 0.934095 | 2.21E-34 | 0.949495 | 1.76E-38 |
| TMEM258 | Up | 0.922449 | 6.81E-32 | 0.954829 | 3.28E-40 |
| ARPC1A | Up | 0.921647 | 9.78E-32 | 0.915133 | 1.60E-30 |
| RNF7 | Up | 0.917059 | 7.19E-31 | 0.902377 | 2.11E-28 |
| IST1 | Up | 0.938832 | 1.58E-35 | 0.934074 | 2.24E-34 |
| POLR2G | Up | 0.926282 | 1.15E-32 | 0.955277 | 2.30E-40 |
| MAGT1 | Up | 0.917851 | 5.14E-31 | 0.948174 | 4.40E-38 |
| PTEN | Up | 0.911206 | 7.79E-30 | 0.923284 | 4.66E-32 |
| PABPC4 | Up | 0.907694 | 3.01E-29 | 0.961267 | 1.34E-42 |
| SIAH1 | Up | 0.912388 | 4.88E-30 | 0.931892 | 7.06E-34 |
| GTF2A2 | Up | 0.892656 | 5.66E-27 | 0.912184 | 5.30E-30 |
| GID8 | Up | 0.910449 | 1.05E-29 | 0.906311 | 5.06E-29 |
| MSL3 | Up | 0.915778 | 1.23E-30 | 0.876814 | 6.45E-25 |
| RPA1 | Up | 0.87846 | 4.07E-25 | 0.91101 | 8.42E-30 |
| KRT7 | Up | 0.864721 | 1.58E-23 | 0.886331 | 4.08E-26 |
| BANF1 | Up | 0.882299 | 1.35E-25 | 0.906249 | 5.18E-29 |
| ZNF706 | Up | 0.899684 | 5.44E-28 | 0.924412 | 2.77E-32 |
| EEF1E1 | Up | 0.885735 | 4.88E-26 | 0.883819 | 8.65E-26 |
| GLRX2 | Up | 0.892732 | 5.53E-27 | 0.929307 | 2.62E-33 |
| NECAP2 | Up | 0.900987 | 3.45E-28 | 0.957914 | 2.62E-41 |
| ALDH18A1 | Up | 0.884734 | 6.59E-26 | 0.908862 | 1.93E-29 |
| BANP | Up | 0.907124 | 3.73E-29 | 0.928036 | 4.92E-33 |
| RPL18 | Up | 0.883239 | 1.03E-25 | 0.924266 | 2.96E-32 |
| SEC61G | Up | 0.883544 | 9.39E-26 | 0.925831 | 1.42E-32 |
| TPD52L2 | Up | 0.907439 | 3.32E-29 | 0.914834 | 1.81E-30 |
| ISG20L2 | Up | 0.886426 | 3.96E-26 | 0.932577 | 4.94E-34 |
| GUK1 | Up | 0.878249 | 4.32E-25 | 0.869454 | 4.68E-24 |
| NME1 | Up | 0.869108 | 5.13E-24 | 0.912454 | 4.76E-30 |
| SLC25A38 | Up | 0.897815 | 1.03E-27 | 0.87457 | 1.20E-24 |
| INTS9 | Up | 0.895158 | 2.51E-27 | 0.914304 | 2.25E-30 |
| RAB8A | Up | 0.877125 | 5.92E-25 | 0.901777 | 2.62E-28 |
| PDIA4 | Up | 0.877035 | 6.06E-25 | 0.922277 | 7.37E-32 |
| HLA | Up | 0.87986 | 2.73E-25 | 0.917397 | 6.23E-31 |
| ELF2 | Up | 0.880354 | 2.37E-25 | 0.851986 | 3.32E-22 |
| MARCKS | Up | 0.870696 | 3.38E-24 | 0.876708 | 6.64E-25 |
| S100A11 | Up | 0.855299 | 1.54E-22 | 0.910071 | 1.21E-29 |
| ADAM10 | Up | 0.861364 | 3.62E-23 | 0.870444 | 3.61E-24 |
| POLR2L | Up | 0.863493 | 2.14E-23 | 0.920406 | 1.70E-31 |
| PDIA6 | Up | 0.862799 | 2.54E-23 | 0.898557 | 8.01E-28 |
| KHDC4 | Up | 0.856792 | 1.09E-22 | 0.822893 | 1.35E-19 |
| VAMP8 | Up | 0.862359 | 2.84E-23 | 0.873782 | 1.48E-24 |
| CTSZ | Up | 0.874346 | 1.27E-24 | 0.94586 | 2.08E-37 |
| SRI | Up | 0.850902 | 4.25E-22 | 0.910658 | 9.66E-30 |
| SSR4 | Up | 0.865767 | 1.21E-23 | 0.891636 | 7.85E-27 |
| FZD1 | Up | 0.846436 | 1.15E-21 | 0.84388 | 2.00E-21 |
| SVIL | Up | 0.853919 | 2.13E-22 | 0.812736 | 8.51E-19 |
| TXNDC9 | Up | 0.860949 | 4.01E-23 | 0.896898 | 1.40E-27 |
| MGLL | Up | 0.851909 | 3.38E-22 | 0.87909 | 3.40E-25 |
| WWC1 | Up | 0.860295 | 4.70E-23 | 0.914638 | 1.97E-30 |
| UBA1 | Up | 0.867717 | 7.35E-24 | 0.867731 | 7.32E-24 |
| TMSB10 | Up | 0.846734 | 1.07E-21 | 0.919557 | 2.46E-31 |
| PSENEN | Up | 0.859162 | 6.18E-23 | 0.932291 | 5.74E-34 |
| INHBC | Up | 0.831955 | 2.35E-20 | 0.857009 | 1.03E-22 |
| PSMB8 | Up | 0.848891 | 6.67E-22 | 0.909501 | 1.51E-29 |
| TNFAIP2 | Up | 0.847277 | 9.54E-22 | 0.879189 | 3.31E-25 |
| HPF1 | Up | 0.870509 | 3.55E-24 | 0.933456 | 3.11E-34 |
| HNRNPA1 | Up | 0.865048 | 1.45E-23 | 0.872835 | 1.91E-24 |
| RPS9 | Up | 0.832909 | 1.94E-20 | 0.8916 | 7.94E-27 |
| ARPC5 | Up | 0.827127 | 6.03E-20 | 0.848975 | 6.55E-22 |
| NDUFS4 | Up | 0.855662 | 1.42E-22 | 0.857497 | 9.20E-23 |
| RPS19 | Up | 0.836435 | 9.52E-21 | 0.89204 | 6.90E-27 |
| FADD | Up | 0.833222 | 1.82E-20 | 0.861579 | 3.44E-23 |
| PTENP1 | Up | 0.841987 | 2.99E-21 | 0.858881 | 6.61E-23 |
| CLP1 | Up | 0.874252 | 1.30E-24 | 0.888363 | 2.19E-26 |
| NAP1L1 | Up | 0.834343 | 1.46E-20 | 0.84111 | 3.61E-21 |
| P4HB | Up | 0.852862 | 2.72E-22 | 0.89778 | 1.04E-27 |
| JPT1 | Up | 0.82045 | 2.12E-19 | 0.893662 | 4.09E-27 |
| PFDN4 | Up | 0.801292 | 5.96E-18 | 0.846771 | 1.07E-21 |
| ATP5IF1 | Up | 0.811904 | 9.84E-19 | 0.806192 | 2.63E-18 |
| DAP | Up | 0.851908 | 3.38E-22 | 0.876203 | 7.64E-25 |
| HSPB1 | Up | 0.817332 | 3.75E-19 | 0.837173 | 8.19E-21 |
| PTPN2 | Up | 0.827312 | 5.82E-20 | 0.87372 | 1.51E-24 |
| FAM98A | Up | 0.834672 | 1.36E-20 | 0.801844 | 5.44E-18 |
| PRDX4 | Up | 0.81006 | 1.36E-18 | 0.88028 | 2.43E-25 |
| APH1A | Up | 0.853469 | 2.36E-22 | 0.877855 | 4.82E-25 |
| WFDC2 | Up | 0.810921 | 1.17E-18 | 0.880837 | 2.07E-25 |
| CD99 | Up | 0.820772 | 2.00E-19 | 0.884098 | 7.97E-26 |
| MYLIP | Up | 0.816544 | 4.32E-19 | 0.860169 | 4.85E-23 |
| POLR2I | Up | 0.835786 | 1.09E-20 | 0.853132 | 2.55E-22 |
| CD58 | Up | 0.801999 | 5.31E-18 | 0.871889 | 2.46E-24 |
| RBPMS | Up | 0.828099 | 5.00E-20 | 0.907721 | 2.98E-29 |
| TBC1D2B | Up | 0.824284 | 1.04E-19 | 0.837498 | 7.66E-21 |
| TRAM2 | Up | 0.813801 | 7.05E-19 | 0.812851 | 8.34E-19 |
| CHFR | Up | 0.814517 | 6.21E-19 | 0.840495 | 4.10E-21 |
| TMED3 | Up | 0.805996 | 2.72E-18 | 0.898691 | 7.65E-28 |
| PYCARD | Up | 0.803336 | 4.25E-18 | 0.883303 | 1.01E-25 |

**Supplementary Table 4.** GS and MM of 116 genes that were used to construct the PPI network. P < 0.05 was considered statistically significant (*). GS, gene significance; HN, hypertensive nephropathy; MM, module membership; PPI, protein-protein interaction.

## Supplementary Table 5

|  |  | **GSE37455** | | **GSE104954** | | **GSE99325** | |
| --- | --- | --- | --- | --- | --- | --- | --- |
| **Category** | **Description** | **logFC** | **P.Value^*^** | **logFC** | **P.Value^*^** | **logFC** | **P.Value^*^** |
| BP | AEROBIC ELECTRON TRANSPORT CHAIN | 0.473496 | 0.000902 | 0.366789 | 0.006368 | 0.421025 | 0.004175 |
| BP | POSITIVE REGULATION OF REACTIVE OXYGEN SPECIES METABOLIC PROCESS | -0.21989 | 0.001399 | -0.22662 | 0.000846 | -0.20873 | 0.002456 |
| BP | POSITIVE REGULATION OF VASCULAR ASSOCIATED SMOOTH MUSCLE CELL PROLIFERATION | -0.21409 | 0.001627 | -0.15963 | 0.043969 | -0.23027 | 0.001582 |
| BP | REGULATION OF NON CANONICAL WNT SIGNALING PATHWAY | -0.26997 | 0.002014 | -0.18031 | 0.049346 | -0.24478 | 0.009756 |
| BP | POSITIVE REGULATION OF CYTOKINE BIOSYNTHETIC PROCESS | -0.31875 | 0.002368 | -0.28724 | 0.005116 | -0.3188 | 0.001865 |
| BP | POSITIVE REGULATION OF SMOOTH MUSCLE CELL PROLIFERATION | -0.1977 | 0.002369 | -0.17634 | 0.005421 | -0.19962 | 0.001409 |
| BP | POSITIVE REGULATION OF REACTIVE OXYGEN SPECIES BIOSYNTHETIC PROCESS | -0.23294 | 0.003007 | -0.24769 | 0.001475 | -0.24769 | 0.001794 |
| BP | ATP SYNTHESIS COUPLED PROTON TRANSPORT | 0.432315 | 0.003797 | 0.370459 | 0.020065 | 0.411277 | 0.004455 |
| BP | TRANSFORMING GROWTH FACTOR BETA PRODUCTION | -0.32281 | 0.003899 | -0.41434 | 0.000226 | -0.25796 | 0.022215 |
| BP | POSITIVE REGULATION OF NITRIC OXIDE METABOLIC PROCESS | -0.25111 | 0.004396 | -0.23148 | 0.007387 | -0.26716 | 0.003543 |
| BP | CYTOKINE METABOLIC PROCESS | -0.2164 | 0.00449 | -0.17888 | 0.031634 | -0.18852 | 0.019941 |
| BP | REGULATION OF REACTIVE OXYGEN SPECIES BIOSYNTHETIC PROCESS | -0.19735 | 0.004694 | -0.20422 | 0.004418 | -0.22068 | 0.002616 |
| BP | TOLL LIKE RECEPTOR 9 SIGNALING PATHWAY | -0.28463 | 0.005042 | -0.19059 | 0.036086 | -0.30149 | 0.002953 |
| BP | REGULATION OF NITRIC OXIDE BIOSYNTHETIC PROCESS | -0.23417 | 0.005085 | -0.23932 | 0.005387 | -0.24653 | 0.004616 |
| BP | NADH DEHYDROGENASE COMPLEX ASSEMBLY | 0.424779 | 0.005574 | 0.399782 | 0.008995 | 0.427295 | 0.004504 |
| BP | TUMOR NECROSIS FACTOR BIOSYNTHETIC PROCESS | -0.28815 | 0.006592 | -0.29539 | 0.004319 | -0.25508 | 0.010537 |
| BP | POSITIVE REGULATION OF TUMOR NECROSIS FACTOR BIOSYNTHETIC PROCESS | -0.32787 | 0.007385 | -0.36337 | 0.001425 | -0.31657 | 0.00601 |
| BP | REACTIVE NITROGEN SPECIES METABOLIC PROCESS | -0.18665 | 0.007833 | -0.18645 | 0.012056 | -0.20835 | 0.005262 |
| BP | REACTIVE OXYGEN SPECIES BIOSYNTHETIC PROCESS | -0.16771 | 0.008013 | -0.15508 | 0.017283 | -0.18403 | 0.006014 |
| BP | ATP SYNTHESIS COUPLED ELECTRON TRANSPORT | 0.354541 | 0.008663 | 0.335629 | 0.020838 | 0.356356 | 0.010146 |
| BP | MITOCHONDRIAL ELECTRON TRANSPORT NADH TO UBIQUINONE | 0.388893 | 0.009348 | 0.391923 | 0.014362 | 0.389849 | 0.009518 |
| BP | RESPONSE TO INTERLEUKIN 6 | -0.21252 | 0.00941 | -0.13698 | 0.045482 | -0.16788 | 0.031512 |
| BP | STRIATED MUSCLE CELL PROLIFERATION | -0.15348 | 0.01025 | -0.14074 | 0.037535 | -0.15229 | 0.008277 |
| BP | MUSCLE CELL PROLIFERATION | -0.14924 | 0.010427 | -0.13796 | 0.024016 | -0.15188 | 0.007069 |
| BP | EMBRYONIC PLACENTA DEVELOPMENT | -0.14415 | 0.010977 | -0.15924 | 0.005181 | -0.13111 | 0.02926 |
| BP | POSITIVE REGULATION OF MYELOID CELL DIFFERENTIATION | -0.19196 | 0.011421 | -0.17614 | 0.015256 | -0.21697 | 0.005543 |
| BP | LYMPHOCYTE APOPTOTIC PROCESS | -0.20716 | 0.011632 | -0.1935 | 0.025058 | -0.1846 | 0.027772 |
| BP | NEGATIVE REGULATION OF LYMPHOCYTE APOPTOTIC PROCESS | -0.24089 | 0.01167 | -0.25746 | 0.015968 | -0.22499 | 0.020824 |
| BP | P38MAPK CASCADE | -0.17596 | 0.011814 | -0.12966 | 0.042381 | -0.16154 | 0.009822 |
| BP | LABYRINTHINE LAYER BLOOD VESSEL DEVELOPMENT | -0.22738 | 0.013326 | -0.26484 | 0.004075 | -0.19742 | 0.045902 |
| BP | OXIDATIVE PHOSPHORYLATION | 0.306508 | 0.013539 | 0.276435 | 0.038411 | 0.311889 | 0.014276 |
| BP | MITOCHONDRIAL RESPIRATORY CHAIN COMPLEX ASSEMBLY | 0.371503 | 0.015725 | 0.370389 | 0.015462 | 0.390172 | 0.009428 |
| BP | ACTIVATION OF MAPKK ACTIVITY | -0.1966 | 0.0158 | -0.16299 | 0.032058 | -0.16886 | 0.030191 |
| BP | RESPIRATORY ELECTRON TRANSPORT CHAIN | 0.318468 | 0.015856 | 0.320605 | 0.022884 | 0.326413 | 0.016617 |
| BP | JNK CASCADE | -0.13907 | 0.015958 | -0.12467 | 0.047542 | -0.13755 | 0.017683 |
| BP | CARDIAC MUSCLE CELL PROLIFERATION | -0.1475 | 0.016792 | -0.15557 | 0.023464 | -0.14883 | 0.023111 |
| BP | PHAGOCYTOSIS | -0.1842 | 0.01762 | -0.17219 | 0.029562 | -0.16632 | 0.034012 |
| BP | ATP BIOSYNTHETIC PROCESS | 0.248637 | 0.018788 | 0.249233 | 0.014779 | 0.24976 | 0.019397 |
| BP | REGULATION OF DEFENSE RESPONSE TO VIRUS BY VIRUS | -0.25607 | 0.021017 | -0.26733 | 0.010036 | -0.21797 | 0.042374 |
| BP | SMOOTH MUSCLE CELL PROLIFERATION | -0.15215 | 0.021368 | -0.137 | 0.03408 | -0.15534 | 0.011539 |
| BP | NEGATIVE REGULATION OF INTERLEUKIN 2 PRODUCTION | -0.23096 | 0.021527 | -0.25043 | 0.014744 | -0.22497 | 0.028571 |
| BP | STRESS ACTIVATED PROTEIN KINASE SIGNALING CASCADE | -0.13116 | 0.025922 | -0.1187 | 0.049407 | -0.12689 | 0.029067 |
| BP | TUMOR NECROSIS FACTOR SUPERFAMILY CYTOKINE PRODUCTION | -0.1829 | 0.026523 | -0.18835 | 0.026631 | -0.1839 | 0.026314 |
| BP | ATRIOVENTRICULAR VALVE DEVELOPMENT | -0.19371 | 0.026779 | -0.22295 | 0.013782 | -0.1688 | 0.039812 |
| BP | POSITIVE REGULATION OF TUMOR NECROSIS FACTOR SUPERFAMILY CYTOKINE PRODUCTION | -0.20638 | 0.02691 | -0.24047 | 0.006748 | -0.20933 | 0.021603 |
| BP | NEGATIVE REGULATION OF PROTEIN BINDING | -0.13045 | 0.027159 | -0.13514 | 0.030148 | -0.16123 | 0.010748 |
| BP | POSITIVE REGULATION OF HEMOPOIESIS | -0.17496 | 0.028148 | -0.16288 | 0.04042 | -0.17529 | 0.029809 |
| BP | REGULATION OF LYMPHOCYTE APOPTOTIC PROCESS | -0.19976 | 0.028442 | -0.19246 | 0.037133 | -0.19238 | 0.044586 |
| BP | REGULATION OF CELL CELL ADHESION | -0.14514 | 0.029336 | -0.13495 | 0.044573 | -0.13427 | 0.044581 |
| BP | POSITIVE REGULATION OF AMYLOID BETA FORMATION | -0.27253 | 0.032475 | -0.30067 | 0.004953 | -0.25122 | 0.031027 |
| BP | PLATELET DEGRANULATION | -0.14331 | 0.033736 | -0.14232 | 0.020018 | -0.13087 | 0.043306 |
| BP | NITRIC OXIDE SYNTHASE BIOSYNTHETIC PROCESS | -0.29071 | 0.033747 | -0.40044 | 0.001119 | -0.29504 | 0.021236 |
| BP | RESPONSE TO ESTROGEN | -0.1318 | 0.036316 | -0.1655 | 0.011648 | -0.16034 | 0.015394 |
| BP | TUMOR NECROSIS FACTOR SECRETION | -0.16383 | 0.037515 | -0.20212 | 0.004009 | -0.17332 | 0.032815 |
| BP | ENDOCYTOSIS+AA3:A56 | -0.10823 | 0.041897 | -0.11197 | 0.039558 | -0.1176 | 0.029899 |
| BP | RECEPTOR MEDIATED ENDOCYTOSIS | -0.11996 | 0.045277 | -0.13903 | 0.025206 | -0.13033 | 0.026476 |
| BP | MYD88 DEPENDENT TOLL LIKE RECEPTOR SIGNALING PATHWAY | -0.19925 | 0.047199 | -0.22164 | 0.039789 | -0.22362 | 0.032222 |
| CC | SMALL RIBOSOMAL SUBUNIT | 0.464512 | 0.000154 | 0.317191 | 0.021982 | 0.468957 | 0.00013 |
| CC | PSEUDOPODIUM | -0.25539 | 0.002245 | -0.31669 | 0.000609 | -0.25738 | 0.002211 |
| CC | LYSOSOMAL LUMEN | -0.24437 | 0.002448 | -0.2112 | 0.02085 | -0.26079 | 0.002136 |
| CC | CYTOSOLIC SMALL RIBOSOMAL SUBUNIT | 0.45175 | 0.002571 | 0.349081 | 0.035489 | 0.463237 | 0.001844 |
| CC | CYTOSOLIC RIBOSOME | 0.427795 | 0.003916 | 0.374524 | 0.024403 | 0.429136 | 0.004981 |
| CC | CYTOCHROME COMPLEX | 0.423643 | 0.004283 | 0.352334 | 0.015207 | 0.437127 | 0.003055 |
| CC | NADH DEHYDROGENASE COMPLEX | 0.457761 | 0.00517 | 0.44558 | 0.009185 | 0.454183 | 0.004814 |
| CC | RESPIRATORY CHAIN COMPLEX | 0.423495 | 0.006915 | 0.378988 | 0.018565 | 0.434094 | 0.005597 |
| CC | CYTOSOLIC LARGE RIBOSOMAL SUBUNIT | 0.443309 | 0.008546 | 0.430268 | 0.020389 | 0.463352 | 0.010295 |
| CC | RESPIRASOME | 0.3818 | 0.009522 | 0.32231 | 0.036749 | 0.38717 | 0.008431 |
| CC | POLYSOME | 0.290403 | 0.009706 | 0.244045 | 0.042761 | 0.297273 | 0.005884 |
| CC | VACUOLAR LUMEN | -0.21335 | 0.011123 | -0.19853 | 0.024092 | -0.23602 | 0.006344 |
| CC | POLYSOMAL RIBOSOME | 0.400666 | 0.012672 | 0.345441 | 0.034356 | 0.384319 | 0.01364 |
| CC | PHAGOCYTIC CUP | -0.27489 | 0.014309 | -0.23868 | 0.025486 | -0.25762 | 0.020769 |
| MF | OXIDOREDUCTASE ACTIVITY ACTING ON A HEME GROUP OF DONORS | 0.420337 | 0.001035 | 0.295115 | 0.017614 | 0.366017 | 0.005562 |
| MF | TRANSMEMBRANE RECEPTOR PROTEIN PHOSPHATASE ACTIVITY | -0.28641 | 0.001147 | -0.30242 | 0.001922 | -0.25577 | 0.002672 |
| MF | ACETYLGALACTOSAMINYLTRANSFERASE ACTIVITY | -0.25339 | 0.003039 | -0.26915 | 0.008285 | -0.26994 | 0.00333 |
| MF | NADH DEHYDROGENASE ACTIVITY | 0.486987 | 0.003412 | 0.454859 | 0.008004 | 0.468911 | 0.003706 |
| MF | PHOSPHATIDYLINOSITOL 3 KINASE BINDING | -0.23033 | 0.005766 | -0.25781 | 0.006038 | -0.19161 | 0.02217 |
| MF | RNA POLYMERASE ACTIVITY | 0.229731 | 0.0064 | 0.236587 | 0.009993 | 0.239198 | 0.003189 |
| MF | INTRAMEMBRANE LIPID TRANSPORTER ACTIVITY | -0.25875 | 0.01075 | -0.1791 | 0.033581 | -0.19083 | 0.044859 |
| MF | SH2 DOMAIN BINDING | -0.20817 | 0.015362 | -0.21518 | 0.004981 | -0.16218 | 0.041546 |
| MF | OXIDOREDUCTASE ACTIVITY ACTING ON NAD P H QUINONE OR SIMILAR COMPOUND AS ACCEPTOR | 0.316408 | 0.030959 | 0.302814 | 0.046841 | 0.313538 | 0.028661 |
| MF | CYCLIN DEPENDENT PROTEIN SERINE THREONINE KINASE REGULATOR ACTIVITY | 0.143181 | 0.046435 | 0.15585 | 0.024772 | 0.156683 | 0.021331 |

**Supplementary Table 5.** GSVA was used to explore POLR2I-related biological function in HN. Only GO terms with P < 0.05 in all datasets are shown (*P < 0.05). BP, biological process; CC, cellular component; FC, fold change; GO, Gene Ontology; GSE, Gene Expression Omnibus Series; GSVA, gene set variation analysis; HN, hypertensive nephropathy; MF, molecular function.

## Supplementary Table 6

|  | **GSE37455** | | **GSE104954** | | **GSE99325** | |
| --- | --- | --- | --- | --- | --- | --- |
| **KEGG pathways** | **logFC** | **P.Value^*^** | **logFC** | **P.Value^*^** | **logFC** | **P.Value^*^** |
| CARDIAC MUSCLE CONTRACTION | 0.253703 | 0.00144 | 0.230452 | 0.006116 | 0.246508 | 0.003814 |
| RNA POLYMERASE | 0.270765 | 0.007539 | 0.281944 | 0.012823 | 0.245245 | 0.011914 |
| RIBOSOME | 0.451035 | 0.008603 | 0.405027 | 0.03125 | 0.460738 | 0.009454 |
| FC GAMMA R MEDIATED PHAGOCYTOSIS | -0.20411 | 0.024269 | -0.21629 | 0.016287 | -0.20844 | 0.01913 |
| LEUKOCYTE TRANSENDOTHELIAL MIGRATION | -0.20575 | 0.0318 | -0.20939 | 0.037464 | -0.21385 | 0.02388 |

**Supplementary Table 6.** GSVA was used to explore POLR2I-related KEGG pathways in HN. Only KEGG pathways with P < 0.05 in all datasets are shown (*P < 0.05). FC, fold change; GSE, Gene Expression Omnibus Series; GSVA, gene set variation analysis; HN, hypertensive nephropathy; KEGG, Kyoto Encyclopedia of Genes and Genomes.

## Supplementary Figure 1


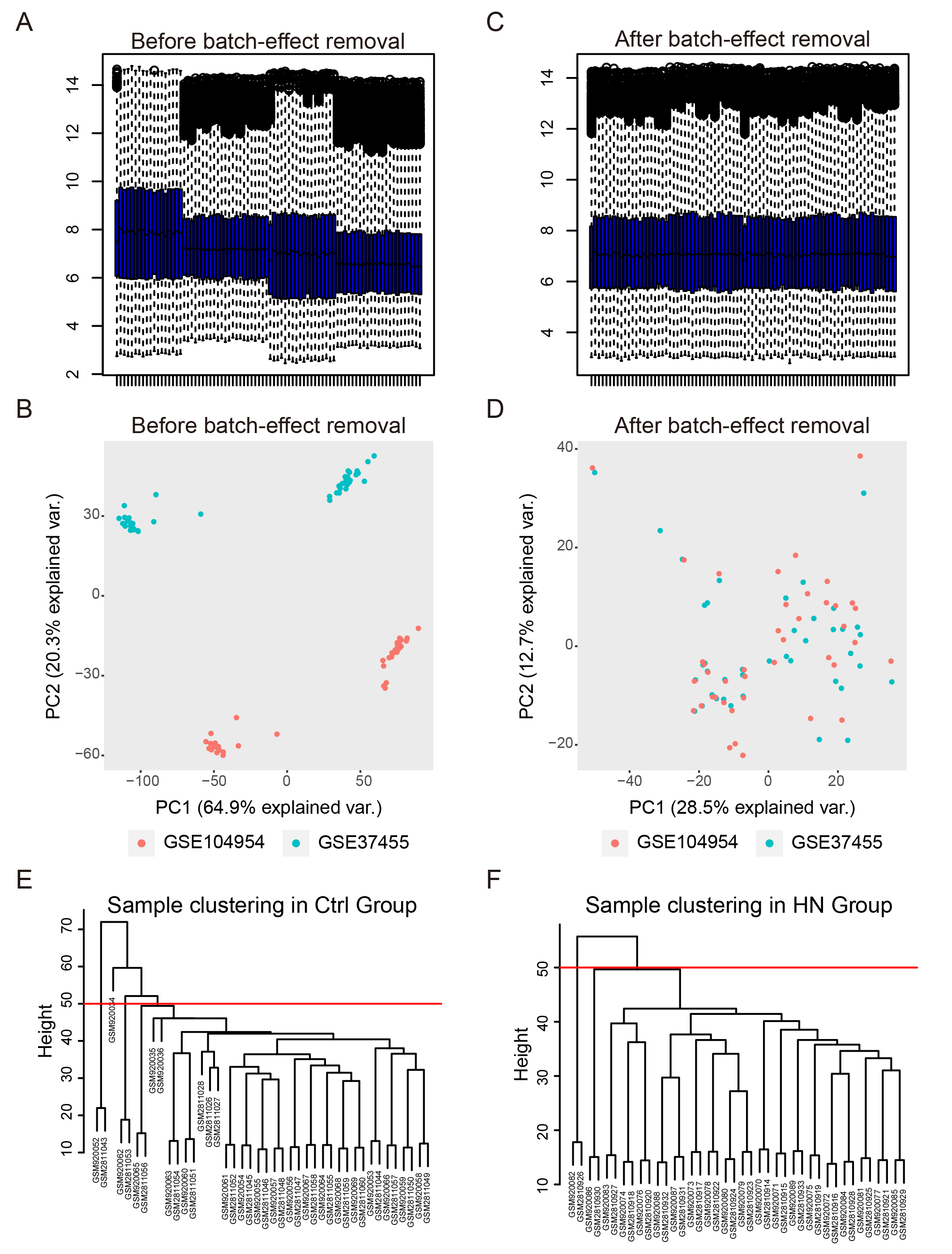


**Supplementary Figure 1.** Data pre-processing. (A-D) The scatter plots based on PCA analysis and the boxplots based on RLE analysis of gene expression profiles without (A, B) and with (C, D) the removal of batch effects. (E, F) Hierarchical clustering of control (E) and HN (F) samples. Red lines on dendrograms refer to the threshold set to identify outliers. We detected 5 outliers in control group and 2 outliers in HN group. GSE, Gene Expression Omnibus Series; HN, hypertensive nephropathy; PCA, principal component analysis; RLE, relative log expression.

## Supplementary Figure 2


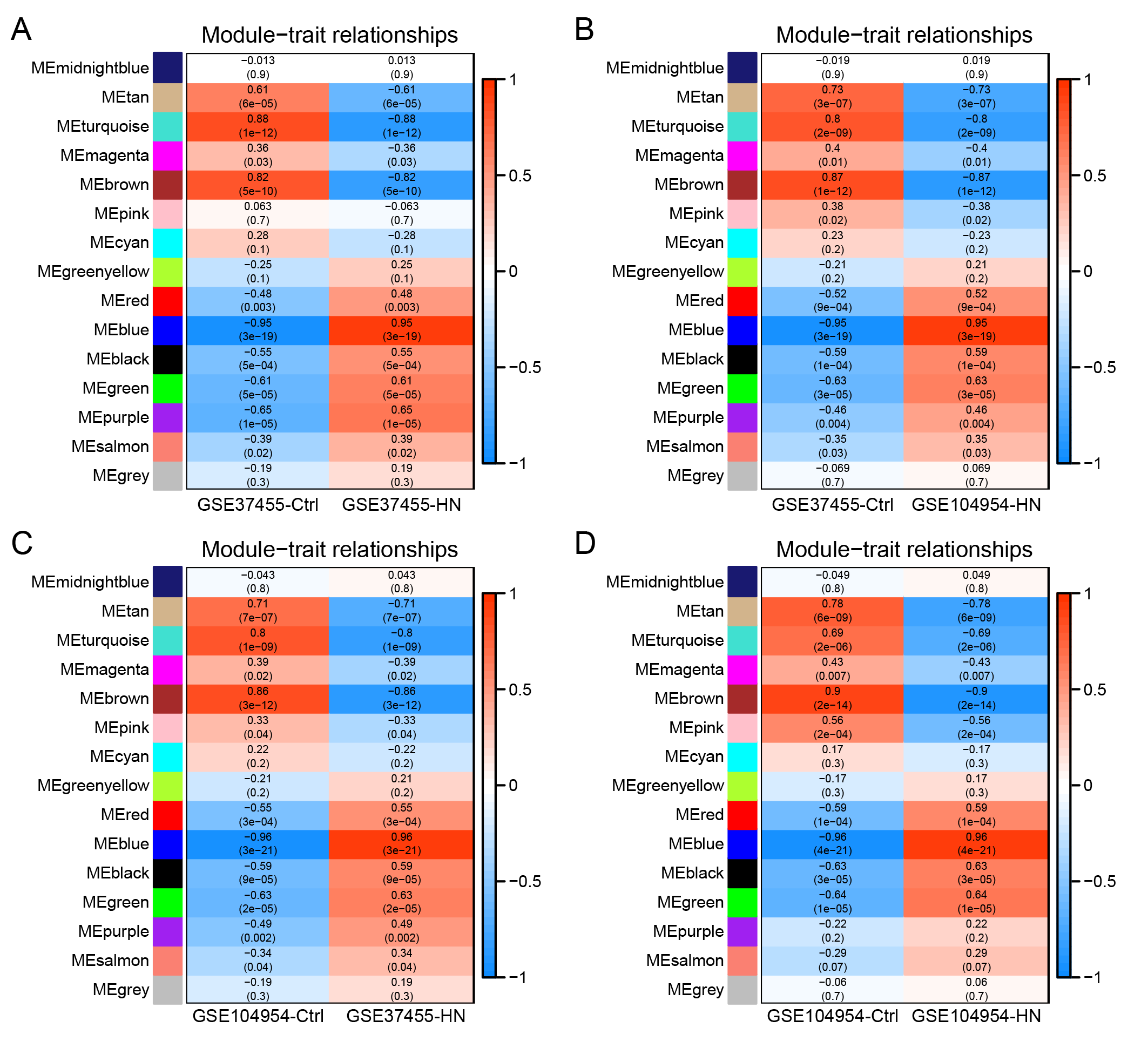


**Supplementary Figure 2.** Module-trait relationships heatmap between each HN subgroup and each control subgroup (A-D). The R-value for each correlation and P-value in parenthesis are shown in the cells. HN, hypertensive nephropathy; ME, module eigengene.
